# M. Lisfranc on Diseased Joints

**Published:** 1826-07

**Authors:** 


27. *Distorted Joints after Sprains, &c.* In the Archives for March of the present year, M. Lisfranc has published a short paper and some cases on the subject of certain consequences which follow sprains and other injuries of joints. This accident, says he, on account of its severity, its frequency of occurrence, its tedious cure, and the weakness which succeeds, is highly deserving of the surgeon's attention. By the employment of antiphlogistics, M. Lisfranc has been able to bring these complaints to a speedy termination. This malady, which M. Lisfranc calls "l'entorse," is one in which the articulating surfaces tend to displace each other, whilst the ligaments and other parts surrounding the articulation, are affected with pain and tension. The articulating surfaces themselves, pressed violently against each other, are inflamed. This affection is more common to the adult age and infancy than to any other. It may occur in any of the articulations, and is more common in the ball and socket-joints than surgeons suppose. He gives two cases where the shoulder-joint was affected in this way, and not being recognized, anchylosis took place. The parts where we most commonly observe it are the ankle, wrist, knee, and elbow. He maintains that what is called a "twist in the loins," is no other than this affection. It may be occasioned by whatever external causes tend to displace the articulating surfaces, without being sufficient to produce actual luxation. The pain accompanying this kind of distortion is very violent. It diminishes soon after the accident, but is renewed again as soon as the fluids are drawn to the part by irritation and inflammation. Sometimes blood, and sometimes other fluids are extravasated into the joints, and exasperate the inflammation. The ligaments even are sometimes torn. There is pain and swelling around the joint, and the heat is much increased The motion of the joint is rendered quite impracticable. Abscesses may form in the neighbourhood of the articulation, and by opening into it cause caries, necrosis, white swelling, &c.

*Treatment.* If the patient be seen early, recourse should be had to refrigerants and astringents, which should be continued for several hours in succession. The joint should also be covered with leeches—40 or 50 in number. If the patient be robust, general bleeding should be combined. If, on the succeeding day, the inflammatory phenomena continue, the local and general bleeding should be repeated, and also on the third day, though to a less extent. After inflammation is subdued, a circular bandage should be applied, but it should be often examined, as the disease is very prone to relapse. When abscesses form they should be opened early, and the antiphlogistic measures carefully pursued, to quell the chronic inflammation. Stimulating liniments and the moxa are only to be employed when all inflammatory symptoms have subsided. Several cases are related in elucidation, but they are not necessary here.

We need only observe that the above is the plan of treatment pursued by all judicious surgeons in this country, after accidents, whether wounds, bruises, or sprains of joints. Perhaps, however, the local bleeding (from the great expense of leeches in this country) is not carried sufficiently far after sprains; while liniments of a stimulating nature, together with bandages, are too early applied. Rest and strict antiphlogistic measures are the surest means of obviating the consequences above portrayed.

